# Glucose‐Sensitive Hydrogel Optical Fibers Functionalized with Phenylboronic Acid

**DOI:** 10.1002/adma.201606380

**Published:** 2017-02-13

**Authors:** Ali K. Yetisen, Nan Jiang, Afsoon Fallahi, Yunuen Montelongo, Guillermo U. Ruiz‐Esparza, Ali Tamayol, Yu Shrike Zhang, Iram Mahmood, Su‐A Yang, Ki Su Kim, Haider Butt, Ali Khademhosseini, Seok‐Hyun Yun

**Affiliations:** ^1^ Harvard Medical School and Wellman Center for Photomedicine Massachusetts General Hospital 65 Landsdowne Street Cambridge MA 02139 USA; ^2^ Harvard‐MIT Division of Health Sciences and Technology Massachusetts Institute of Technology Cambridge MA 02139 USA; ^3^ Biomaterials Innovation Research Center Division of Engineering in Medicine Brigham and Women's Hospital Harvard Medical School Cambridge MA 02139 USA; ^4^ State Key Laboratory of Advanced Technology for Materials Synthesis and Processing Wuhan University of Technology 122 Luoshi Road Wuhan 430070 China; ^5^ Department of Chemistry Imperial College London South Kensington Campus London SW7 2AZ UK; ^6^ Department of Biological Sciences Korea Advanced Institute of Science and Technology Daejeon 34141 South Korea; ^7^ School of Engineering University of Birmingham Birmingham B15 2TT UK; ^8^ Wyss Institute for Biologically Inspired Engineering Harvard University Boston MA 02115 USA; ^9^ Department of Physics King Abdulaziz University Jeddah 21589 Saudi Arabia; ^10^ Department of Bioindustrial Technologies College of Animal Bioscience and Technology Konkuk University Hwayang‐dong, Gwangjin‐gu Seoul 143‐701 South Korea

**Keywords:** diagnostics, fiber optics, glucose sensing, hydrogels, light transmission

## Abstract

**Hydrogel optical fibers** are utilized for continuous glucose sensing in real time. The hydrogel fibers consist of poly(acrylamide‐*co*‐poly(ethylene glycol) diacrylate) cores functionalized with phenylboronic acid. The complexation of the phenylboronic acid and *cis*‐diol groups of glucose enables reversible changes of the hydrogel fiber diameter. The analyses of light propagation loss allow for quantitative glucose measurements within the physiological range.

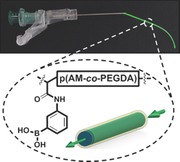

Continuous glucose monitoring (CGM) devices measure the concentration of glucose in interstitial fluid.^1^ These devices aim to provide real‐time, long‐term measurements that can also be used with insulin pumps to form an automated feedback loop, which can suspend insulin delivery when hypoglycemia is developing.^2^ CGM, however, does not completely solve issues associated with low patient compliance. At least 3–4 fingerstick blood tests per day must be performed to calibrate CGM blood glucose concentration, and the implantation of the sensor probe and bulkiness of the device present discomfort to patients.^3^ They also have signal drift due to the instability of electrochemical reactions in vivo, and are associated with time lags, in addition to the high cost of the sensor replacement every 3–7 days.^4^

Optical glucose sensors are attractive detection platforms for the continuous quantification of glucose concentration.[[qv: 1b,5]] Optical sensors offer advantages over electrochemical assays since they can be constructed to be label‐free, provide real‐time continuous monitoring for long periods of time, are immune to electromagnetic interference, and can be calibrated internally.^6^ One of the promising approaches for optical glucose sensors is to covalently incorporate glucose‐sensitive chelating agents such as phenylboronic acid (PBA) derivatives^7^ into matrices such as for micro and nanostructures including holographic thin films,^8^ crystalline colloidal arrays,^9^ plasmonic nanoantennas,^10^ Fabry–Perot cavities,^11^ fluorescent dyes,^12^ and quantum dots.^13^ Optical monitoring systems have also been developed in the form of solid‐state optodes that report on the glucose concentration via refractive index (RI) changes.^14^ Recently, solid‐state optical fiber glucose sensors comprising fluorescent diboronic acid receptors have been clinically tested for continuous intravascular glucose monitoring.^15^ However, optical sensors utilizing solid‐state materials (e.g., silica) are not fully compatible with biological systems for implantation in vivo.[[qv: 1b,16]] Solid‐state optical fibers may cause infection and immune reactions (foreign body responses) at an implanted site, resulting in inflammation and discomfort to patients.^17^ This necessitates the development of biocompatible implantable biosensors.

Hydrogels have been utilized in biomedicine due to their tunable optical and mechanical properties.^18^ For example, cell‐seeded hydrogel waveguides have been developed for implantation.^19^ Recently, core‐clad waveguides have been fabricated from poly(ethylene glycol) (PEG) derivatives and silk.^20^ Among these polymer‐based sensors, hydrogel optical fibers are a promising technology for quantifying glucose for biomedical applications due to their biocompatibility and capability to incorporate functional groups for sensing.^19^ For instance, optical polymer fibers based on fluorescent sensing have been reported for quantitative glucose measurements.^12^ However, photobleaching of the fluorophore, and variations in the illumination source and output caused over/underestimation of the glucose concentration in vivo. Additionally, this technology was not applicable to individuals with skin pigmentation, light scattering from the tissue, and was affected by epidermal thickness.^21^ The stiff hydrogel fibers have been fabricated from PEG‐diacrylate (PEGDA) (700 Da), which was not compatible with sensing mechanisms based on volumetric change‐induced quantitative measurements in hydrogels.[[qv: 20a]]

Here, we create hydrogel optical fibers having a poly(acrylamide‐*co*‐poly(ethylene glycol) diacrylate) p(AM‐*co*‐PEGDA) core and a Ca alginate cladding. 3‐(acrylamido)phenylboronic acid (3‐APBA) molecules were covalently incorporated into the core for sensing glucose. We investigated the changes of the physical and optical properties of the hydrogel fiber sensors in response to glucose. Quantitative readouts were obtained from measuring the changes in the intensity of transmitted light through the hydrogel optical fibers. The main advantages of hydrogel optical fiber sensors over current technologies include: (i) flexibility for potential implantation, (ii) reproducibility to sense glucose concentrations in real time within the glucose concentration range in diabetes (normal: 4.2–6.4, diabetic: 3.0–20.0, diagnosis >7.0 mmol L^−1^), (iii) prolonged continuous sensing, and (iv) biocompatibility. Hydrogel optical fibers may be inserted subcutaneously to monitor the concentration of glucose in interstitial fluid.

The light‐guiding efficiency of an optical fiber is determined by light loss at the interface between the core material and surrounding cladding. To maximize light propagation, the core should possess ha igher RI than the cladding, and both should have high light transmission. The light attenuation and RIs of p(PEGDA), p(AM‐*co*‐PEGDA), and Ca alginate were measured. **Figure** **1**a shows the light transmission at 532 nm through hydrated p(PEGDA) matrix (1 × 1 × 1 cm^3^, 10–90 vol%). The minimum of the data points indicated polymerization‐induced phase separation due to the immiscibility of PEGDA with water (20–60 vol%) (Figure S1, Supporting Information).^22^ The optical properties of p(AM‐*co*‐PEGDA) hydrogels were not affected by the AM or PEGDA (700 Da) concentration. After UV‐induced polymerization, concentrations of p(AM‐*co*‐PEGDA) (10–90 mol%) hydrogels had >94% light transmission (Figure 1a inset). Figure S2 in the Supporting Information illustrates the images of AM, PEGDA, p(PEGDA), and p(AM‐*co*‐PEGDA). UV–vis spectra of p(AM‐*co*‐PEGDA) and p(PEGDA) (70–90%, v/v) and hydrogels showed minimal light attenuation in the visible spectrum (Figure 1b). Light was not attenuated at wavelengths longer than 400 nm. Increase in the concentration of the Na alginate from 1 to 4 wt% enhanced light attenuation (Figure 1c).

**Figure 1:**
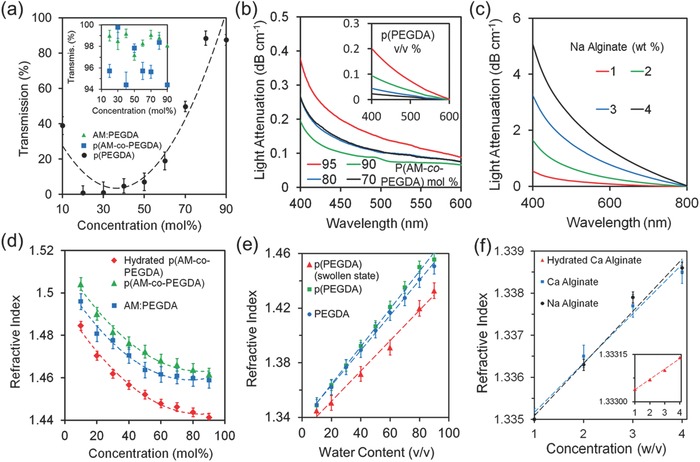
Optical properties of p(PEGDA), p(AM‐*co*‐PEGDA), and Ca alginate hydrogels at 24 °C. a) Light transmission (532 nm) of p(PEGDA), p(AM‐*co*‐PEGDA), hydrogels as a function of precursor concentration from 0 to 90 mol% in DI water. Error bars represent three independent samples (*n* = 3). b) Light attenuation of p(AM‐*co*‐PEGDA) hydrogel at different crosslinking densities. The inset shows light attenuation of p(PEGDA) hydrogel at different concentrations. c) Absorption spectra of Na alginate solutions at different concentrations. d) RIs of AM and p(AM‐*co*‐PEGDA) (2:3, AM wt/vol% dilution in DI water) at different relative concentration ratios. e) RIs of PEGDA and p(PEGDA) at as a function of diluted monomer concentration. f) RIs of Na alginate and Ca alginate hydrogels at different concentrations (1–4 wt%). The inset shows the RI change of hydrated Ca alginate as a function of Na alginate concentration.

Figure 1d shows the RIs of AM:PEGDA monomer mixture, and p(AM‐*co*‐PEGDA) hydrogels. As the concentration of acrylamide increased, the RI decreased from 1.50 to 1.46. Figure S3 in the Supporting Information shows the RIs of AM at different concentrations. PEGDA was co‐polymerized with AM to obtain flexible hydrogels. The RIs of p(PEGDA) hydrogels showed la inear relationship with increasing monomer concentration (Figure 1e). At the minimum monomer concentration, the RI of p(PEGDA) hydrogel (10 mol%, *n* = 1.351) was higher than the RI of Ca alginate hydrogel with maximum concentration (alginate, 4 wt%, *n* = 1.339) (Figure 1f). The RIs of the hydrated p(AM‐*co*‐PEGDA), p(PEGDA) hydrogel and Ca alginate were significantly lower than their nonhydrated states. Based on the optical properties of the core and cladding materials, p(AM‐*co*‐PEGDA), p(PEGDA), and Ca alginate precursors were optimized to construct the hydrogel optical fibers.

Monomer solution was injected into a poly(vinyl chloride) (PVC) tube that served as a mold (**Figure** **2**a). The monomer solution within the mold was exposed to a UV light for crosslinking (Figure 2b). Scheme S1 in the Supporting Information shows the polymerization process of the monomer solution. The hydrogel fiber core was ejected from the mold by applying water pressure (Figure 2c). The hydrogel core was submerged in a Na alginate solution, followed by a CaCl_2_ solution (100 mmol L^−1^) to form a Ca alginate hydrogel cladding. The fabrication process was ≈5 min, the synthesized hydrogel was immediately ready for use as an optical fiber by coupling with laser light (Figure 2d). The incorporation of fluorescent dye‐conjugated red and green beads to the core and the cladding enabled the visualization of the hydrogel fiber assembly (Figure 2e). This fabrication process allowed for synthesizing a range of hydrogel fiber cores with different thicknesses from 200 µm to 2.0 mm (Figure 2f). Hydrogel fibers having 200 µm diameters are thinner than the sensing probe diameters of commercial CGM systems (250–400 µm) (Table S1, Supporting Information). The contraction of the hydrogel fibers upon polymerization ranged from 3.21 to 6.49%. The thickness of Ca alginate cladding (50–100 µm) increased 3.69 ± 3.67 µm as the concentration of Na alginate increased from 1.0 to 4.0 wt% (Figure 2f inset). To analyze the mechanical properties of the hydrogel fibers, the tensile strain was measured (Figure 2g). As the diameter decreased from 2.0 mm to 200 µm, the tensile strain of p(AM‐*co*‐PEGDA) (97:3 mol%) and p(PEGDA) fibers increased from 0.13 to 0.74 mm mm^−1^ and from 0.13 to 0.47 mm mm^−1^, respectively. As compared to p(PEGDA), the tensile strain of the fibers (*Ø* = 200 µm) was higher in p(AM‐*co*‐PEGDA), where AM comonomer rendered the fiber elastic. As the p(AM‐*co*‐PEGDA) diameter decreased from 2.0 mm to 200 µm, the maximum load at maximum tensile stress increased from 2.8 to 9.5 MPa and modulus decreased from 27 to 20 MPa (Figure S4, Supporting Information). Figure S4 in the Supporting Information shows the maximum load of PEGDA and tensile stress of the p(AM‐*co*‐PEGDA) fibers and the experimental setup for the tensile stress measurements.

**Figure 2:**
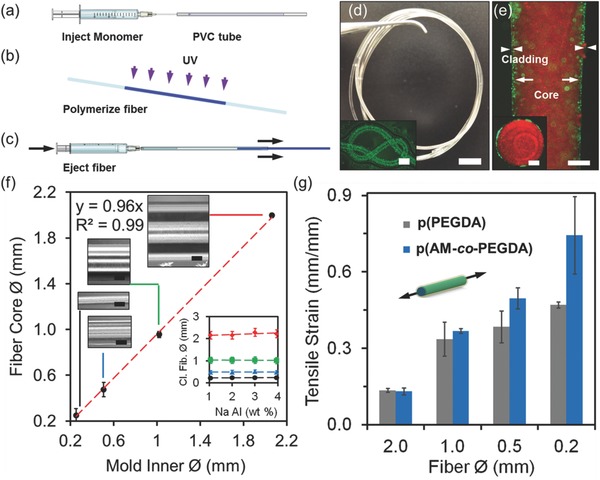
Fabrication and characterization of hydrogel optical fibers. a–c) Fiber fabrication. d) Photographs of the fabricated hydrogel fibers. Scale bar = 5 mm. The inset shows the flexibility of the hydrogel fiber knot having green fluorescent beads. Scale bar = 500 µm. e) A fluorescent image of the hydrogel fiber showing core (red) and cladding (green). Scale bar = 500 µm. The inset shows the fiber cross‐section. Scale bar = 250 µm. f) Fiber core diameter as a function of inner diameter of the PVC mold. Scale bars = 200 µm. The inset graph shows the change of the cladded fiber diameter as a function of Na alginate concentration. g) Characterization of the tensile strain and values of the p(AM‐*co*‐PEGDA) and p(PEGDA) hydrogel fibers with varying diameters. Error bars represent three independent samples (*n* = 3 in f,g).

The effectiveness of Ca alginate claddings for light guiding were tested by measuring the reduction in scattered light intensity over the hydrogel fiber lengths. Laser light (532 nm, 1 mW) was focused on the tip of hydrogel fibers (length = 6 cm) with and without cladding in air (**Figure** **3**a). Noncladded hydrogel cores guided the light with significant scattering (Figure 3b). However, cladded hydrogel fibers efficiently guided the light over 20 cm (Figure 3c). The analysis of light intensity profile of scattered light showed that the light loss of the cladded hydrogel fiber ranged from 1 to 6 dB cm^−1^; however, the bare fiber core light loss was within 2–11 dB cm^−1^. Light propagation loss at 532 nm was comparable to 491 nm light (Figure S5, Supporting Information). Figure 3d shows change in light attenuation for different fiber diameters. As compared to the thinner fibers, 2.0 mm core showed higher light transmission, which may be attributed to the longer ray propagation distances before reflecting off light from the core‐air interface. Furthermore, light dispersion over fiber distance for the cladded fiber was consistent as compared with the noncladded fiber, which had light intensity fluctuations.

**Figure 3:**
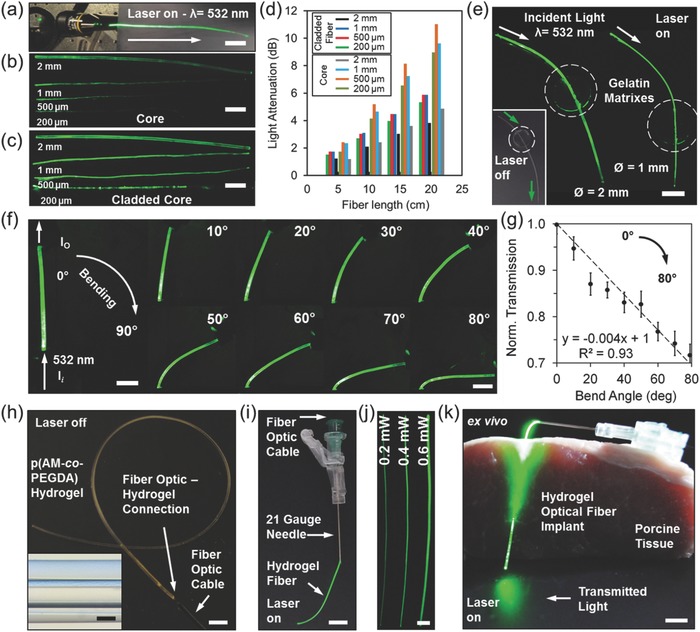
Light propagation in hydrogel optical fibers. a) Coupling of laser light (λ = 532 nm) to the cladded fiber tip to guide light. b) Light attenuation in noncladded hydrogel fibers at different thicknesses in air. c) Light guidance in cladded hydrated fibers with different thicknesses in air. d) Scattered light intensity along hydrogel fiber with bare core and cladding. e) Light transmission of hydrogel fibers through gelatin phantom tissues. Scale bar = 1 cm. The inset shows the sandwiched hydrogel fiber when the laser is off. f) Photographs of the fibers angularly rotated (0° to 80°) hydrogel fibers. Scale bar = 5 mm. g) Macroscopic bending loss as a function of bend angle. Error bars represent three independent samples (*n* = 3). h) Coupling the p(AM‐*co*‐PEGDA) hydrogel fibers with fiber optic cables. Scale bar = 5 mm. The inset shows the optical fiber coupled to p(AM‐*co*‐PEGDA) fiber (insertion = 1 cm). Scale bar = 100 µm. i) The integration of hydrogel fibers with needles for creating insertable devices. Scale bar = 5 mm. j) Hydrogel fibers in hypodermic needles at different light intensities. Scale bar = 2 mm. k) The implantation of hydrogel optical fibers in porcine tissue. Scale bar = 3 mm.

The light transmission of the hydrogel fibers were tested through gelatin matrices as phantom tissues. Gelatin matrices were used to visualize the light transmission throughout the hydrogel optical fibers. The hydrogel fibers (*Ø* = 1.0–2.0 mm) were inserted into tissue‐mimicking phantom samples and the fibers were illuminated with a laser light source (λ = 532 nm) (Figure 3e). Light attenuation in fibers due to contact with the gelatin matrices was less than 10%. The light attenuation in the p(PEGDA) hydrogel fibers were also evaluated by bending tests. One end of a hydrogel fiber was kept mounted on a stand, and the hydrogel fiber was illuminated with a continuous wave laser light (*I*_i_, λ = 532 nm, 1 mW). The intensity of light at the output (*I*_o_) was measured as the tip of the fiber was bent from 0° to 80° (Figure 3f). As the bend angle increased, the intensity of light due to bending decreased 30% at 80° (Figure 3g). The decrease in the intensity of the transmitted light due to bending might be attributed to the increase in the surface roughness, distortions, or fractures in the Ca alginate cladding.

The hydrogel fibers were connected to solid‐state step‐index multimode fiber optic cables to launch light. Solid‐state optical fiber and hydrogel optical fiber connection was created by co‐polymerizing the monomer solution and a silica fiber in a PVC mold (Figure 3h). The solid‐state optical fiber within the hydrogel optical fiber had a core diameter of 100 µm and NA of 0.37. The inset in Figure 3h shows a silica optical fiber coupled to a p(AM‐*co*‐PEGDA) hydrogel fiber. The hydrogel–silica fiber connection was stable when the hydrogel was fully hydrated. The resulting hydrogel optical fiber was integrated within a 21 gauge needle (inner *Ø* = 514 µm) for implantation in tissue (Figure 3i). The light intensity of the hydrogel fibers in the needles could be finely controlled (Figure 3j). The resulting hydrogel optical fiber was injected within porcine tissue as deep as 3 cm and was retractable after implantation (Figure 3k). Figure S6 in the Supporting Information shows a threaded hydrogel optical fiber in the porcine tissue. The implanted hydrogel fiber can deliver light into deep tissues for application in photodynamic therapy and biosensing.

**Figure** **4**a shows a hydrogel optical fiber with a tunable p(AM‐*co*‐PEGDA‐*co*‐3‐APBA) core and a Ca alginate cladding. Scheme S2 in the Supporting Information shows the synthetic scheme of p(AM‐*co*‐PEGDA‐*co*‐3‐APBA). As glucose molecules diffuse into the hydrogel fiber and complex (1:1) with PBA derivatives, the Donnan osmotic pressure of the system increases. Variation in the osmotic pressure, hence the hydrogel density changes the RI that affects light propagation through the hydrogel fiber (Figure 4b–d). The measurement of *I*_o_ as a function of tuning the core RI can be utilized as a sensor to quantify analyte concentrations.

**Figure 4:**
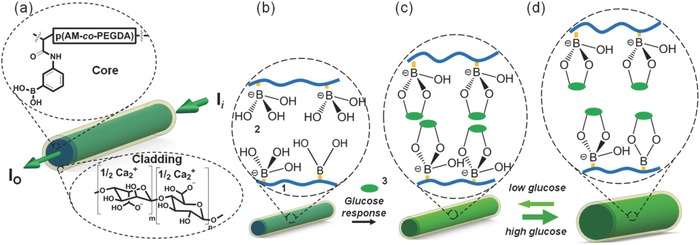
Design of the glucose‐sensitive hydrogel optical fibers. a) Structural composition of the glucose‐sensitive p(AM‐*co*‐PEGDA‐*co*‐3‐APBA) fiber core cladded with Ca alginate. b) The hydrogel matrix is functionalized with 3‐APBA. (1) PEG‐crosslinked polyacrylamide hydrogel, (2) 3‐APBA in charged tetrahedral state, (3) glucose. c) The PBA derivative binds *cis* diols of glucose molecules and changes the RI of the hydrogel fiber. d) The increase in the concentration of the glucose molecules can be quantified by measuring the variation in the intensity of the output light.

PBA derivatives complex with carbohydrates through their *cis*‐diol groups (Scheme S3, Supporting Information). The binding between the anionic boronate species and *cis*‐diol groups of glucose molecules is covalent. For example, polymers fabricated with dynamic covalent boronic esters were shown to undergo reversible bonding with self‐healing properties.^23^ The complexation equilibrium depends on the p*K*_a_ of the copolymer. At acidic pH (<7.0), the uncharged trigonal planar form of the PBA does not readily complex with glucose.^24^ However, above the p*K*_a_ point of the PBA (≈8.6–8.8), its charged tetrahedral state reversibly binds to glucose. Hydrogel fibers consisting of 3‐APBA (15 mol%) and PEGDA (3–4 mol%) were fabricated and tested in the presence of glucose in PBS (pH 7.4, 100.0 mmol L^−1^, 24 °C). The complexation of the glucose with 3‐APBA lowers the apparent p*K*_a_ of the p(AM‐*co*‐PEGDA‐*co*‐3‐APBA) system. The increase in the concentration of the anionic boronate species increases the free mixing energy and the hydrophilicity.^25^ The formation of boronate anions upon glucose‐3‐APBA complexation increases the Donnan osmotic pressure of the hydrogel fiber.^26^

The glucose sensing properties of hydrogel optical fibers were investigated. To test the 3‐APBA‐glucose complexation dynamics, glucose (100 mmol L^−1^) and 3‐APBA (15 mol%) were used. Previously in acrylamide‐based hydrogel sensors, 20 mol% 3‐APBA was found to be the optimum in hydrogel‐based glucose sensors.^27^ Figure S8 in the Supporting Information shows that light attenuation did not change between precursors (AM:PEGDA:3‐APBA, 77/3/20 mol% in DMSO) before and after filtering (0.22 µm), indicating that 3‐APBA completely dissolves in DMSO. The attenuation decreased 4.6% after polymerization in DMSO. After polymerization of the precursor in aqueous solution, the light attenuation decreased by 7.5%. In comparison to 3‐APBA dissolved in DMSO, light attenuation of the precursor in DI water decreased ≈24.3%. Hence, ≈15 mol% was the maximum solubility of 3‐APBA in the present system in aqueous solutions. The hydrogel fibers containing 3.0 mol% PEGDA was found to be optimum, expanding the hydrogel 8.7% in diameter (**Figure** **5**a). The fibers were fully swollen in glucose‐free buffer solutions before the sensing experiments. The complexation of charged tetrahedral 3‐APBA with *cis* diols of glucose molecules reached equilibrium in 40 min. Hydrogel fibers without the functional group 3‐APBA had 0.3% expansion due to nonspecific interaction with glucose. At higher concentrations of PEGDA (3.5, 4.0 mol%), the increase in the crosslinking density decreased the elasticity of the hydrogel optical fiber, limiting the fiber expansion to ~6.7%, in the presence of glucose (100 mmol L^−1^). However, at lower concentrations of PEGDA (1.0, 2.0 mol%), the Donnan osmotic pressure effect due to ionic strength of the buffer did not significantly expanded (0.9 and 1.0%) the hydrogel fiber diameter. As the concentration of glucose within the physiological range was increased from 4.0 to 12.0 mmol L^−1^, cross‐section area of p(AM‐*co*‐PEGDA‐*co*‐3‐APBA) hydrogel fibers expanded by 6% over 1 h (Figure 5b). Figure S9 in the Supporting Information shows the hydrogel fiber diameter expansion in the presence of glucose (0–20 mmol L^−1^). Increase in the concentration of the glucose in p(AM‐*co*‐PEGDA) without the functional group 3‐APBA did not significantly change the hydration state of the fiber over 1 h.

**Figure 5:**
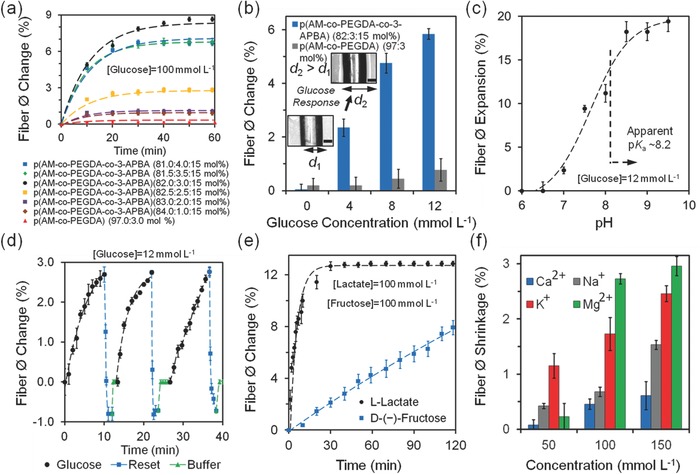
Quantification of glucose concentrations with p(AM‐*co*‐PEGDA‐*co*‐3‐APBA) optical fibers at pH 7.4 at 24 °C. Optical fibers were fully swollen during the experiments. a) Time‐lapse measurements of the expansion of hydrogel fiber diameter (*Ø* = 1 mm) in the presence of glucose (100 mmol L^−1^) and control experiments fitted with the exponential decay equation, where the decay constant α is 9.1 × 10^−4^ s^−1^. b) The change in the diameter of the hydrogel fibers as the glucose concentration is increased. Scale bar = 500 μm. c) pH‐dependent fiber expansion (12.0 mmol L^−1^). d) pH dependency of the sensor in sensing glucose (pH 7.4, 12.0 mmol L^−1^, 24 °C). The sensor fiber diameter was returned to its original size by using acetate buffer (pH 4.6), followed by PBS rinse. e) Sensor response to d‐(−)‐fructose and l‐lactate (100 mmol L^−1^) over 1 h. f) The effect of metal ions (ionic strength) in fiber shrinkage. Error bars represent three independent samples (*n* = 3 in a–f).

The area expansion of the fiber cross‐section and the concentration of glucose at the boundaries *C*_∞_ had a linear relation. Figure 5b shows the relation between Δ*Ø* and *C*_∞_ from 0 to 10 mmol L^−1^, followed by saturation of the expansion. To model the time‐dependency of the fiber expansion with respect to the concentration at the boundaries, it was assumed that fiber matrix only had charged tetrahedral state of 3‐APBA with a saturation trend. By considering a uniform distribution of glucose inside the hydrogel fiber, the diffusion dynamics can be approximated as:

(1) $$\frac{dC\left(t\right)}{dt}\;=\;\frac{4D}{{\left(\emptyset /2\right)}^{2}}\left[C_{\infty }\;-\;C\left(t\right)\right]$$

where *C*(*t*) is the time‐dependent concentration of glucose in the hydrogel fiber, and *D* is the diffusion constant. The solution of Equation (1) is an exponential function:

(2) $$C\;\left(t\right)\;=\;C_{\infty }\;\left(1\;-\;e^{\;-\alpha t}\right)$$

where α is a decay constant:

(3) $$\alpha \;=\;\frac{4D}{{\left(\emptyset /2\right)}^{2}}$$

The solution for the hydrogel fiber diameter expansion Δ*Ø* is:

(4) $$\Delta \emptyset \left(t\right)\;=\;\Delta \emptyset_{\infty }\sqrt{1\;-\;e^{-\alpha t}}$$

where Ø_∞_ represents the expanded diameter after infinite time. The decay constant α describes the affinity of boronic acid‐*cis* diol complexation and is correlated with the diffusion constant of the glucose–hydrogel fiber system. The α coefficient extracted from the fitted diameter expansion was 37 × 10^−5^ s^−1^, and the diffusion constant *D* was 23.75 µm^2^ s^−1^. This diffusion is ~4 times faster than rhodamine B (model diffusion system) due to its lower molecular weight and hydrophilicity (Figure S7, Supporting Information). Another limiting factor that contributes to the curve saturation is the elastic limit of the hydrogel fiber during expansion. The complexation between the 3‐APBA and *cis* diols of glucose molecules depends on the apparent p*K*_a_ value of the hydrogel system. To measure the apparent p*K*_a_ value of the hydrogel fibers, the pH was varied at a fixed glucose concentration. As the pH of the glucose solution (12.0 mmol L^−1^) was increased up to 9.5, the fiber diameter expanded by 19% (Figure 5c). Henderson–Hasselbalch equation was used to retrieve the apparent p*K*_a_ value of the p(AM‐*co*‐PEGDA‐*co*‐3‐APBA) hydrogel fiber,

(5) $$\emptyset_{\mathrm{shift}}\;=\;\frac{\Delta \emptyset }{\left(10^{\left(pK_{a}\;-\;\mathrm{pH}\right)}\;+\;1\right)}$$

where *Ø*_shift_ is the fiber diameter shift, Δ*Ø* is the difference between the maxima and minima diameter points, and p*K*_a_ is the acid dissociation constant. The measured apparent p*K*_a_ value of the hydrogel fiber was 8.2. At this p*K*_a_ value, the degree of glucose bound to tetrahedral state of the 3‐APBA with degrees of ionization of 12.1 (pH 7.0), 21.5 (pH 7.5), and 46.2 (pH 8.0), followed by reaching equilibrium.

When immersed in a buffered glucose solution (pH 7.4, 12 mmol L^−1^), the hydrogel fiber containing 3 mol% PEGDA and 15 mol% APBA formed reversible covalent bonds with glucose molecules producing a diameter expansion of 2.5% around 9 min (Figure 5d). For a typical diabetic patient, the required readout rate for a shift of glucose concentration from 8.0 to 15.0 mmol L^−1^ is 0.078 mmol L^−1^ min^−1^. The hydrogel optical fiber sensor provided a readout rate of 1.33 mmol L^−1^ min^−1^, which was 17‐fold higher than the required speed. When the glucose containing PBS buffer was replaced with acetate buffer (pH 4.6), the hydrogel fiber contracted 3.5%. The shrinkage in hydrogel fiber can be attributed to the decrease of pH of the system below the apparent p*K*_a_ value of the hydrogel fiber. The charged tetrahedral state transformed to uncharged trigonal planar form and released bound glucose molecules. Addition of PBS at 7.4 returned the hydrogel fiber diameter to its original position. Figure 5d shows three consecutive glucose addition, buffering and sensor resetting cycles. Sensor reset was achieved in 1 min and no hysteresis was recorded during repeat measurements of glucose.

Potential interferents of 3‐APBA‐glucose complexation include carbohydrates and l‐Lactate. As the buffer solution was replaced with buffered fructose solution (100 mmol L^−1^, pH 7.4), the hydrogel fiber diameter linearly expanded by 8% over 2 h, showing a slower binding rate than glucose (Figure 5e). These results agree with the previous studies that showed higher affinity of boronic acid to fructose than glucose under physiological conditions.^28^ Fructose blood concentrations in diabetic patients and healthy human subjects are 12.0 ± 3.8 and 8.1 ± 1.0 µmol L^−1^, respectively.^29^ The blood fructose concentration corresponds to 0.096% expansion over 2 h in hydrogel fibers. Another interferent, l‐lactate through its α‐hydroxy acids competitively binds to 3‐APBA.^30^ When the buffer solution was replaced with buffered lactate solution at pH 7.4, the hydrogel fibers expanded 13% over 15 min. As compared to glucose and fructose, rapid expansion of the hydrogel fibers in the presence of l‐lactate can be attributed to rapid diffusion induced by its small molecular weight (*M*_W_: 90 g mol^−1^) and high affinity through its α‐hydroxy acid. The concentration of blood l‐lactate in healthy resting adults is 0.36–0.75 mmol L^−1^.^31^ This concentration corresponds to 0.047%–0.097% hydrogel fiber expansion. Hence, the competitive binding of both fructose and lactate are below 1% error in hydrogel fiber swelling.

The effect of unspecific charge interactions of metal ions (ionic strength) with p(AM‐*co*‐PEGDA‐*co*‐3‐APBA) dynamics were also evaluated. As the concentration of metal ions (Na^+^, K^+^, Ca^2+^, and Mg^2+^) were increased from ion‐free solution to 150 mmol L^−1^, the hydrogel fiber diameter shrunk 1.5, 2.4, 0.6 and 2.9%, respectively (Figure 5f). The concentration ranges of Na^+^, K^+^, Ca^2+^, and Mg^2+^ ions in blood are 136.0–145.0, 3.5–5.0, 1.1–1.4, and 0.8–1.2 mmol L^−1^, respectively.^32^ The error of glucose concentration readings in the presence of ion variation corresponds to Na^+^ (−1.435%), K^+^ (−0.097%), Ca^2+^ (−0.002%), and Mg^2+^ (−0.023%) ions. The combined maximum ionic strength effect of blood electrolytes corresponds to ≈1.55% shrinkage in the hydrogel fiber. In the cases of hypo/hypernateremia, the maximal hydrogel diameter changes would be ≈1.55 ± 0.14%. This shrinkage could be attributed to the nonspecific charge interactions between the metal ions and hydrogel fiber matrix, resulting in decrease in Donnan potential. Additionally, the hydrogel is a filter for soluble high molecular weight molecules including proteins and glycated proteins. The effect of temperature variation in sensing was investigated from 24 to 37 °C in p(AM‐*co*‐PEGDA‐*co*‐3‐APBA) hydrogel fibers within the physiological concentrations (4–16 mmol L^−1^) in (100 mmol L^−1^, pH 7.4). The error was 0.7% at 37 °C as compared to 24 °C (Figure S10, Supporting Information). Hence, temperature changes did not significantly affect the fiber diameter expansion and glucose measurements.

Reversible complexation of 3‐APBA and *cis* diols of glucose molecules enabled the use of hydrogel fibers as dynamic sensors. Glucose concentrations ranging from 1.0 to 12.0 mmol L^−1^ were introduced to the p(AM‐*co*‐PEGDA‐*co*‐3‐APBA) hydrogel fiber (**Figure** **6**a). Sensor saturation response was ≈20 min for each increased concentration value. The hydrogel fibers expanded 6% as the concentration of glucose was incrementally increased up to 12.0 mmol L^−1^ (arrows show concentration changes). As the concentration of glucose was decreased, the hydrogel fibers returned to their original diameter sizes. The decomplexation time was ≈30 min for each glucose concentration decrease.

**Figure 6:**
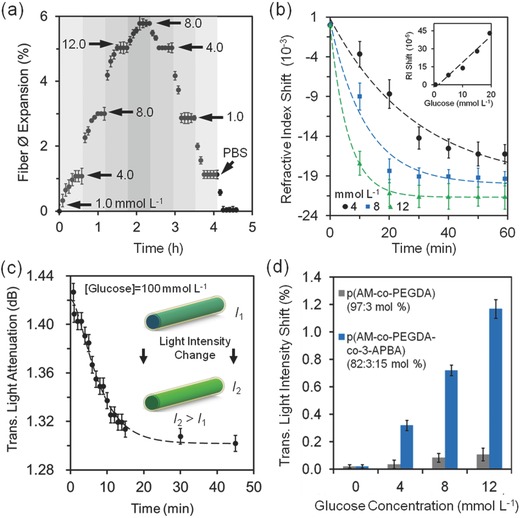
p(AM‐*co*‐PEGDA‐*co*‐3‐APBA) hydrogel fibers as a glucose sensor (pH 7.4, 1.0–12.0 mmol L^−1^, 24 °C). The optical fibers were fully swollen during the experiments. a) Reusability of the hydrogel fibers in sensing glucose. b) The change in the RI of the hydrogel fibers in the presence of physiological glucose concentrations. The inset shows the RI of glucose with increasing concentration. c) Transmitted light attenuation across the hydrogel fiber as function of time boronic acid‐glucose *cis* diol binding (100 mmol L^−1^). d) Transmitted light intensity across the hydrogel fiber measured in different glucose concentrations (4.0–12.0 mmol L^−1^), showing a decrease in light scattering with increasing glucose concentration. Error bars represent three independent samples (*n* = 3 in a–d).

As the concentration of glucose was increased up to 12 mmol L^−1^ over 1 h, the RI of the p(AM‐*co*‐PEGDA‐*co*‐3‐APBA) core decreased from by 0.02 units due to the increase in Donnan osmotic pressure (Figure 6b). At high concentrations of glucose (20–50 mmol L^−1^), the decrease in the RI of the p(AM‐*co*‐PEGDA‐*co*‐3‐APBA) core decreased 0.04 from an original value of 1.383. Saturation of sensor response over 30 min indicated fully complexed 3‐APBA‐glucose molecules. The expansion of the hydrogel fibers due to 3‐APBA‐glucose complexation counteracts ionic strength effect. The ionic strength effect of Na^+^ ions (137 mmol L^−1^), and to a lesser extent to K^+^ ions (2.7 mmol L^−1^) and at pH 7.4, shifted the RI by 0.025.

To use the p(AM‐*co*‐PEGDA‐*co*‐3‐APBA) hydrogel fibers as a RI‐based optical sensor, the changes in the intensity of transmitted light across the fiber were measured by extracting the attenuation between the input and output light. A CW laser (532 nm, 1 mW) was used to illuminate hydrogel fibers swollen in different glucose concentrations. To test the 3‐APBA‐glucose complexation dynamics, we used 100 mmol L^−1^ glucose. At sensor response equilibrium in 45 min, the transmitted light intensity across the fiber decreased 8.3% (Figure 6c). The change in the transmitted light was correlated with the concentration of the glucose. The propagation loss (γ) across an optical fiber can be expressed as:^33^

(6) $$\gamma \;=\;\frac{4\sigma^{2}h^{2}}{\beta \left(\emptyset \;+\;\frac{2}{p}\right)}\;=\;\frac{\sigma^{2}k_{0}^{2}hI_{n}\Delta n^{2}}{\beta }$$

where σ is the roughness of core‐cladding interface, *k*_0_ is the free space wavenumber, β is the modal propagation constant, Δ*n* is the difference between the RIs of the hydrogel core and Ca alginate cladding, and *h* and *p* are the transverse propagation constants in the core and cladding, respectively; and *I*_n_ is normalized field intensity at the core‐cladding interface. The parameters σ, *k*_0_, β, *h*, and *p* were assumed to be constant. Upon boronic acid‐*cis* diol glucose complexation, an increase in Donnan osmotic pressure increases *Ø* and decreases Δ*n*. Hence, the change in both *Ø* and Δ*n* decrease the propagation loss (Equation (6)), thus producing an increase in the intensity of transmitted light across the hydrogel fiber. Δ*n* is proportional to the time‐dependent concentration of the glucose *C*(*t*). Hence propagation loss (γ) can be expressed as:

(7) $$\gamma \propto \frac{\sigma^{2}k_{0}^{2}h\;I_{n}\;C^{2}}{\beta }\propto 10\;\log\frac{I_{i}}{I_{o}}$$

(8) $$\gamma \;=\;\tau {\left[C_{\infty }\left(1\;-\;e^{-\alpha t}\right)\right]}^{\;2}$$

where τ is the proportionality constant. The diffusion constant *D* is correlated with the decay constant α (Equation (3)), which can be utilized to determine the dynamics of γ in time. For long periods of time (>30 min), *e* ^−*αt*^ approaches zero and the sensor response equilibrates (Equation (8)). At short periods of time, Equation (8) can be expressed in a linear manner:

(9) $$\sqrt{\gamma }\;=\;\tau C_{\infty }\alpha t$$

where the slope is given by *τC*_∞_α. Hence, *C*_∞_ can be inferred at short periods of time (<5 min) by measuring the rate of γ in time. Within the physiological range, the transmitted light intensity across the hydrogel optical fiber shifted 1.2% as the concentration of glucose was increased from 4 to 12 mmol L^−1^, showing a sensitivity of 1.2 mmol L^−1^ (Figure 6d). The absorption of light by glucose in the physiological range was a minor contributor the signal shift (Figure S11, Supporting Information).

The growth and viability of the cells in the presence of hydrogel optical fibers has been further assessed. We cultured NIH‐3T3 fibroblasts in multiwall plates in the presence of 3‐APBA functionalized fibers (PEGDA, p(AM‐*co*‐PEGDA) and p(AM‐*co*‐PEGDA‐*co*‐3‐APBA)) (length = 1 cm). The metabolic activity of cells was assessed using PrestoBlue assay to investigate the effect of the used fibers on cellular growth and function (**Figure** **7**a). The results showed no significant difference between the growth of cells interfaced with fibers fabricated from various constituents of the 3‐APBA functionalized fibers and the control without fiber. Cellular viability was also assessed by using a Live/Dead Assay Kit over 7 days of culture (Figure 7b,c). Most of the cells were stained as green (live) and some cells were red (dead) confirming the high cellular viability (>95%) (Figure 7c).

**Figure 7:**
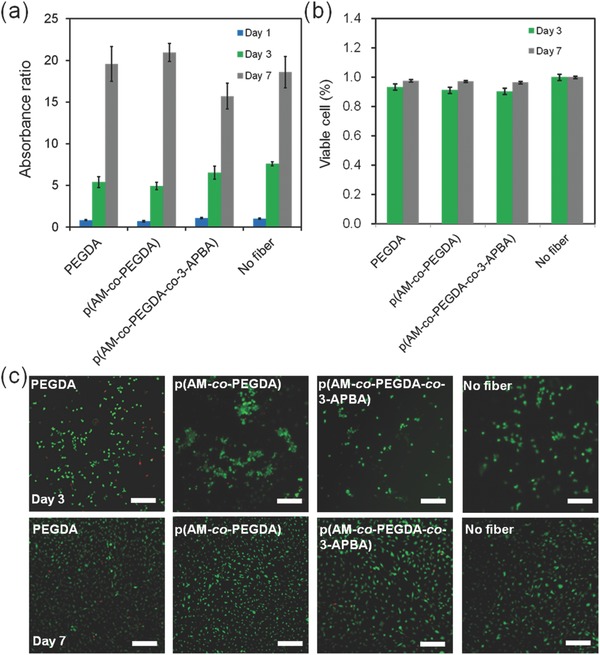
Biological study of NIH‐3T3 fibroblasts for fiber samples: PEGDA, p(AM‐*co*‐PEGDA), p(AM‐*co*‐PEGDA‐*co*‐3‐APBA), and no fiber. a) Cellular metabolic activity measured with PrestoBlue assay and compared to control confirming normal proliferation of cells exposed to the 3‐APBA functionalized fibers. b,c) LIVE/DEAD assay for assessing cellular viability on day 3 and day 7, where live cells are stained in green and dead cells in red. Scale bar = 50 µm. (*n* = 3 in a,b)

As compared to fluorescent boronic acid receptors, the measurement of light transmission based sensing is not prone to photobleaching.[[qv: 13c]] In contrast to the photonic crystal sensors that only expand in *z*‐direction, hydrogel optical fibers expand isotropically.^26^ Unlike enzymatic reactions involving glucose oxidase or hexokinase‐glucose‐6‐phosphate dehydrogenase,^34^ 3‐APBA molecules competitively bind to *cis* diols of carbohydrates (e.g., fructose) as well as lactate through its α‐hydroxy acids.^30^ While 3‐APBA was demonstrated as a model ligand for glucose in the present work, selectivity can be improved by co‐polymerizing tertiary/quaternary amine monomers (e.g., (3‐acrylamidopropyl)trimethylammonium) with PBA derivatives.^35^ Alternatively, PBA derivatives such as 2‐acrylamido‐5‐fluorophenylboronic acid,^8^ 2‐(acrylamido)phenylboronate,^36^ 4‐vinylphenylboronic acid (4‐VPBA),^37^ or their copolymers^38^ can be used to enhance the selectivity of the hydrogel fiber sensors to glucose. Electron‐withdrawing substituents may increase the sensor response by decreasing the p*K*_a_ of PBA.^26^ One of the factors that affected the sensing time was the diffusion rate of the glucose molecules into the optical hydrogel fibers. Other phenylboronic acid functionalized high‐surface area copolymer geometries such as microgel particles^11^, ^39^ and porous thin films^40^ can be utilized to improve the responsivity to glucose within the hydrogel optical fiber platform for achieving faster equilibrium. Furthermore, co‐monomers can be utilized with volume resetting agents to provide linear response to glucose.^41^ Additionally, the boric acid group of the hydrogel optical fibers is pH dependent. However, for diagnostics applications, the pH of the interstitial fluid is tightly regulated by bicarbonate buffer (pH 7.4). When acid–base homeostasis is not balanced, for example, in acidosis, interstitial pH can decrease below 7.35; and in alkalosis, pH of the interstitial fluid may exceed 7.45. Hence, coupling the phenylboronic acid sensor with a pH sensor may allow the compensation of the pH error in measurements.^42^ To improve the mechanical properties of hydrogel optical fibers, highly stretchable alginate–polyacrylamide hydrogels may be adopted.^43^ Additionally, the development of robust cladding polymers that can bind to the core covalently will improve the efficiency of light transmission in vivo.^44^

Intensity‐based readout in quantitative measurements is associated with potential light loss through fiber bending. This may be mitigated by employing sophisticated photonic sensing schemes such as whispering‐gallery mode analysis[[qv: 20a]] and diffraction gratings.^45^ The glucose‐induced swelling of the hydrogel matrix with embedded gratings can change lattice spacing and/or RI to produce Bragg peak shifts for quantitative analysis.^46^ Additionally, the presented hydrogel optical fibers can be functionalized with chelating agents, proteins, oligomers, nanopores, and channel‐based membranes to be responsive to a wide range of analytes for sensing and drug delivery applications.^47^ Hydrogel optical fibers show potential for label‐free optical sensing toward continuous in vivo glucose monitoring systems for diabetes patients at clinical and point‐of‐care settings.

## Supporting information

As a service to our authors and readers, this journal provides supporting information supplied by the authors. Such materials are peer reviewed and may be re‐organized for online delivery, but are not copy‐edited or typeset. Technical support issues arising from supporting information (other than missing files) should be addressed to the authors.

Supplementary
