# Supplementary material for: Glucose‐Sensitive Hydrogel Optical Fibers Functionalized with Phenylboronic Acid
Source: Adv Mater. 2017 Feb 13;29(15):1606380. doi: 10.1002/adma.201606380 (PMC5921932; doi:10.1002/adma.201606380)
Supplement: Supplementary file 1 — Supplementary [file ADMA-29-na-s001.pdf]

Copyright WILEY-VCH Verlag GmbH & Co. KGaA, 69469 Weinheim, Germany, 2017.

ADVANCED MATERIALS

Supporting Information

for *Adv. Mater.*, DOI: 10.1002/adma.201606380

Glucose-Sensitive Hydrogel Optical Fibers Functionalized with Phenylboronic Acid

Ali K. Yetisen, Nan Jiang, Afsoon Fallahi, Yunuen Montelongo, Guillermo U. Ruiz-Esparza, Ali Tamayol, Yu Shrike Zhang, Iram Mahmood, Su-A Yang, Ki Su Kim, Haider Butt, Ali Khademhosseini, and Seok-Hyun Yun**

Copyright WILEY-VCH Verlag GmbH & Co. KGaA, 69469 Weinheim, Germany, 2016.

Supporting Information

Glucose-Sensitive Hydrogel Optical Fibers Functionalized with Phenylboronic Acid

Ali K. Yetisen Nan Jiang, Afsoon Fallahi, Yunuen Montelongo, Guillermo U. Ruiz-Esparza, Ali Tamayol, Yu Shrike Zhang, Iram Mahmood, Su-A Yang, Ki Su Kim, Haider Butt, Ali Khademhosseini, and Seok-Hyun Yun**

Contents

1. Materials

2. Equipment

3. Experimental Section

Formulation of the monomer solution

Optical characterization of hydrogel fibers

Tensile testing of the hydrogel fiber

Image processing

Preparation of phantom gel samples

Fiber Optic connection to hydrogel optical fibers

Fabrication of glucose-responsive hydrogel fibers

Preparation of D-(+)-glucose, D-(−)-fructose, L-lactate, and metal ion samples

Measurements of analyte concentrations in hydrogel fibers

Diffusion analysis in hydrogel fibers

Variation of temperature in hydrogel optical fibers

Cell Viability Assessment

Cell Proliferation Assay

Supporting Information Figures

Materials

All chemicals were of analytical grade and used without further purification. Poly(ethylene glycol) diacrylate (PEGDA) (mw: 700 Da), acrylamide (AM) (98%), 2-hydroxy-2-methylpropiophenone (2-HMP) (97%), poly(methylhydrosiloxane) (PDMS, average Mn 1,700-3,200), gelatin (10 wt%, porcine skin, gel strength 300 g Bloom), 3-(acrylamido)phenylboronic acid (3-APBA) (98%), D-(+)-glucose (99.5%), D-(−)-fructose (99%), sodium L-lactate, sodium chloride (99.5%), potassium chloride (99%), calcium chloride (98%), magnesium chloride (98%), phosphate buffered saline (PBS) tablets (10 mmol L^{-1} phosphate buffer, 2.7 mmol L^{-1} potassium chloride and 137 mmol L^{-1} sodium chloride, pH 7.4), sodium phosphate monobasic NaH_2PO_4 (lower pH), rhodamine B (95%) (sodium phosphate dibasic, acetate buffer (acetic acid-sodium acetate, pH 4.6), hydrochloric acid (37%), sodium alginate, rhodamine B were purchased from Sigma-Aldrich. Tris base was purchased from Fisher Scientific. Green and red fluorescent microbead solutions were purchased Createx colors (East Granby, CT). A fluorescence-based LIVE/DEAD® viability/cytotoxicity assay kit consisting of intracellular green-fluorescent calcein acetoxymethyl and red-fluorescent ethidium homodimer-1 stains was purchased from Invitrogen (USA).

Equipment

Step-index multimode fiber optic cables (core=100 µm, NA: 0.37, FC connector) were purphased from Thorlabs. Hypodermic needles (21 G 1 TW, 0.8 mm × 25 mm, SafetyGlide) were purchased from Becton Dickinson. Poly(vinyl chloride) (PVC) tubes (Masterflex Tygon S3) with different inner diameters (ID): 0.5-2.0 mm) were purchased from Cole Parmer. The UV light source (Spectroline) was purchased from Spectronics Corporation. A digital refractometer (300053) was purchased from Sper Scientific. A hotplate was purchased from

Barnstead /Thermolyne. A continuous wave laser (λ=532 nm) equipped with a power supply (FPU 35) was purchased from Laser Quantum finnesse. A 4× lens (plan N, ∞/-/FN 22) with a NA size of 0.10 mm was purchased from Olympus. A hand-held optical power meter (Model 1918-R) was purchased from Newport Corporation. A digital single-lens reflex camera (D90, 12.3 MP) and a lens (AF-S DX 18-105 mm f/3.5-5.6G ED VR) were purchased from Nikon. An optical microscope (IX51) with phase contrast and fluorescence imaging was purchased from Olympus. A charge-coupled device (CCD) (2MP) color digital microscope camera was purchased from Spot RT3. A mechanical analyzer (model 5524) was purchased from Instron (Canton, MA). ImageJ (v 1.50a) and Orifin were used from image processing and data plotting. SolidWorks (2016 SP1, Dassault Systèmes) was used for creating schematics. A UV-visible spectrometer (UV-2450) was purchased from Shimadzu Corporation (Japan). A drop shape ultrasonic cool mist humidifier with 2.3 gallon output per day was purchased from Crane. An optical spectrophotometer having 0.1 nm resolution was obtained from Thorlabs. An inverted fluorescence microscope TE 2000-U was purchased from Nikon instruments Inc., USA. Fluorescence-based LIVE/DEAD® viability/cytotoxicity assay kit and PrestoBlue® assay were purchased from Invitrogen, CA. A microplate reader was purchased from Biotek, USA.

Experimental Section

Formulation of the monomer solution: The precursor solution consisted of acrylamide (AM) (10-97 mol%) and poly(ethylene glycol) diacrylate (PEGDA) (3-90 mol%, 700 Da), which were mixed with 2-hydroxy-2-methylpropiophenone (2-HMP) (2 vol%) in deionized water. The dilution of the monomer solution was varied from 10-90 vol%. For the glucose formulation containing 3-(acrylamido)phenylboronic acid (3-APBA) (15 mol%), the dilution was 2:3 (AM wt%: DI water vol%). The monomer solution was filtered through 0.45 μm pores. Fluorescent hydrogels were prepared by using a fluorescent microbead solution diluted

with DI water (1:9, v/v). Diluted microbead solutions (50 µL, red, green) were copolymerized with PEGDA solutions (1 mL) and Na alginate and $CaCl_2$ solutions, respectively, to form and clad hydrogel fibers. Supporting Information provides a list of materials.

Optical characterization of hydrogel fibers: Refractive index (RI) measurements were carried out by a digital refractometer. For light attenuation test, an optical setup consisting of a laser guided the light into the optical fiber. A continuous wave laser (λ=532 nm, 491 nm) equipped with a power supply produced 1 mW light intensity. The light was focused on the tip of the hydrogel fiber using a 4× lens (plan N, ∞/-/FN 22) with a numerical aperture (NA) of 0.10. The laser light intensities were measured by an optical power meter. Photographs of optical fibers were taken by a digital single-lens reflex camera (12.3 MP) using a lens (AF-S DX 18-105 mm f/3.5-5.6G ED VR) operated at ISO 1600, 1/1500 speed, and F3.5. The hydrogel optical fibers were imaged by an optical microscope (phase contrast, fluorescence) equipped with an objective (4×, NA: 0.13) and charge-coupled device (CCD) color digital camera (2 MP). Images were imported into ImageJ and intensity values were extracted along the optical fiber image based on the intensity input from the optical power meter measurements. The transmission values were measured by illuminating a polymer cube (1×1×1 cm^3) with a continuous wave laser light (λ=532 nm) and collecting the transmitted light with an optical power meter. Supporting Information provides the equipment list.

Tensile testing of the hydrogel fiber: The national standard used for the tensile strain measurements was American Section of the International Association for Testing Materials (ASTM) D3039. Hydrogel fibers (length=10 cm, Ø=2.0, 1.0, 0.5, and 0.2 mm) were kept in DI water prior to testing. Their mechanical properties were measured using an Instron analyzer in the tensile mode (load cell 10 N). The fibers were sandwiched between the grips with an initial distance of 5 cm and were stretched at a strain rate of (0.2 mm min^{-1}). The hydrogel fibers were kept moist using an ultrasonic humidifier during testing.

Image processing: Photographs of optical fibers were taken by a digital single-lens reflex camera (12.3 MP) using a lens (AF-S DX 18-105 mm f/3.5-5.6G ED VR) operated at ISO 1600, 1/1500 speed, and F3.5. The images of hydrogel optical fibers were taken by an optical microscope (phase contrast, fluorescence) equipped with a lens (4×, NA: 0.13) and charge-coupled device (CCD) color digital camera (2 MP). Images were imported into ImageJ and intensity values were extracted along the optical fiber image based on the intensity input from the optical power meter measurements. The transmission values were measured by illuminating a polymer cube (1×1×1 cm^3) with a continuous wave laser light (λ=532 nm) and collecting the transmitted light with an optical power meter.

Preparation of phantom gel samples: Phantom matrixes were prepared from gelatin (porcine skin, gel strength: 300 g Bloom). Gelatin (10 wt%) was dissolved in DI water and heated up to 70 °C as it was stirred until it was fully dissolved. Before solidification, the gelatin solution (6 mL) was poured into a Petri dish (Ø= 3.5 cm) and allowed to solidify at 4 °C for 15 min. To measure the bending loss, hydrogel fibers (Ø=1, 2 mm) were sandwiched between two phantom samples (thickness = 0.6 cm) in the center. The light was guided into the hydrogel fibers using a 532 nm laser light. The bending loss was measured by a powermeter.

Fiber Optic connection to hydrogel optical fibers: The hydrogel fibers were integrated with step-index multimode fiber optic cables to efficiently deliver light. The fiber optic cable had a core diameter of 100 µm and NA of 0.37 with a FC connector.

Fabrication of glucose-responsive hydrogel fibers: Poly(vinyl chloride) (PVC) tube as fiber molds (10 cm, inner diameter (Ø): 0.5-2.0 mm) was attached to a ruler in horizontal and leveled position. The monomer solution (800 mL) was injected into the fiber mold using a syringe. The monomer solution was polymerized by exposing the mold to UV light (365 nm, 5 mW cm^{-2}) for 3 min. The crosslinked hydrogel fiber core was extracted from the tube by applying water pressure using a syringe. The core was rinsed with ethanol and water (1:1, v/v)

and placed in PBS. The cladding was prepared by immersing the hydrogel fiber cores in Na alginate (1-4 wt%) for 3 s and dried for 3 min. The fibers were submerged in calcium chloride solution ($CaCl_2$, 100 mmol L^{-1}) for 1 min to form a Ca alginate cladding.

Preparation of D-(+)-glucose, D-(−)-fructose, L-lactate, and metal ion samples: Stock solutions (100 mL) of phosphate-buffered saline (PBS pH 7.4, ionic strength=150 mmol L^{-1}), a buffer solution used to maintain cell metabolic activity, were prepared in the absence and presence of glucose (20.0-100.0 mmol L^{-1}), respectively. The buffer solution containing glucose was serially diluted with the buffer solution (glucose-free) to prepare glucose concentrations ranging from 1.0 to 14.0 mmol L^{-1} with a constant ionic strength. The buffers solutions were immediately used after preparation and fresh solution were prepared for each trial. For pH titration experiments, sodium phosphate monobasic and sodium phosphate dibasic was used to obtain pH buffers (6.0-9.5, 150 mmol L^{-1}). The concentrations of D-(−)-fructose and L-lactate solutions were 100.0 mmol L^{-1}. Metal ion solutions (50.0-150.0 mmol L^{-1}) in tris buffers (pH 7.4, 100 mmol L^{-1}) were prepared from NaCl, KCl, $CaCl_2$, and $MgCl_2$.

Measurements of analyte concentrations in hydrogel fibers: Hydrogel fibers (Ø=200 μm, 500 μm, 1.0 mm and 2.0 mm, l=5 mm) were placed on a Petri dish under a phase-contrast microscope. The hydrogel fibers were immersed in PBS (pH 7.4) and allowed to stabilize at 24 °C. The PBS solution was replaced by glucose, D-(−)-fructose, and L-lactate solutions (4.0-12.0 mmol L^{-1}, 100.0 mmol L^{-1}, 3 mL) and the fiber diameters were measured with 1 min increments starting from the lowest concentration over 2 h and reaching signal equilibrium. For the reusability experiments, glucose solution was replaced by acetate buffer (pH 4.6) for 3 min to decrease the pH and release the glucose molecules from 3-APBA. The hydrogel fibers were rinsed with PBS solution three times for 5 min before being immersed into the higher concentration of glucose solution. Control experiments were carried out by measuring the concentrations of glucose (4.0-12 mmol L^{-1}) and glucose in p(PA-*co*-PEGDA-*co*-3-APBA) (82:3:15 mol%) using a UV-Vis spectrophotometer within the range of 200-800 nm calibrated

with PBS. In metal ion measurements, the hydrogel fibers were placed in a Petri dish containing tris buffer (tris base-HCl, pH 7.4, 100 mmol L^{-1}) and allowed to equilibrate for 3 min at 24 °C. Metal ion solutions were introduced to the system from lower to higher concentrations and allowed to equilibrate for 1 min. After each measurement, the metal ions were rinsed with tris buffer for 3 min while being stirred.

Diffusion analysis in hydrogel fibers: Rhodamine B (0.1 mmol L^{-1}) was allowed to diffuse into the hydrogel fibers (Ø=2.0 mm, 20-80 wt%) over 5 min. After every minute, the hydrogel fibers were dried (24 °C, RH: 60%) and hydrogel fibers were cut across the transverse plane. The intensity profile of rhodamine B diffused into the hydrogel was imaged using a fluorescence microscope and quantified using ImageJ.

The diffusion of rhodamine B (100 µmol L^{-1}, Mw=479 g mol^{-1}) into the hydrogel fibers was measured as a model using fluorescence microscopy. The fluorescence intensity had a linear relation with the concentration of rhodamine B. By correlating the measured intensity with the concentration, diffusion profile at the fiber was obtained. Figure S7 shows the diffusion of rhodamine B into the hydrogel fibers (PEGDA 4:1, 2:1, 1:1 vol%) over 3 min, and also shows time-lapse diffusion plots of the diffusion of rhodamine dye into hydrogel fibers and the microscopy photographs. Generally, the diffusion of molecules in aqueous solutions can be described with random molecular motion. As the hydrogel matrix was homogeneous, the molecular motion was not directional. Nevertheless, there is a net transfer from high to low dye concentrated region due to the random motion. This mass transfer can be described in terms of diffusion equation (heat equation). By analyzing the diffusion process at the periphery of the fiber, the cylindrical structure was simplified as a 1D problem. By fitting the concentration profile with the Equation S1, the diffusion coefficients of the different crosslinked hydrogels were extracted. The diffusion coefficients for hydrogels containing 1:2, 1:1, 4:1 vol% PEGDA were 4.5, 2.4, and 0.9 $\mu m^2 s^{-1}$, respectively. The inset in Figure S7 shows the cross-sectional images of hydrogel fibers with diffusing inward

gradient of rhodamine B extending from the peripheries toward the center of the fiber. The solution for the 1D diffusion equation in space and time C(x, t), with a constant concentration at the boundaries C_∞ and an initial C_0 is:

$$\frac{C(x,t)-C_\infty}{C_0-C_\infty} = \mathrm{erfc}\left(\frac{x}{2\sqrt{Dt}}\right) \qquad (S1)$$

where D is the diffusion coefficient.

Variation of temperature in hydrogel optical fibers: p(AM-*co*-PEGDA) and p(AM-*co*-PEGDA-*co*-3-APBA) hydrogel fibers (Ø=1.0 mm) were cut in 1 cm lengths, and immersed in glucose solution (4-16 mmol L^{-1}) in tris buffers (pH 7.4, 100 mmol L^{-1}). The measurements were carried out in a Petridish on a hotplate connected to a thermometer (24 °C - 37°C). The fiber diameter changes were measured under an optical microscope.

Cell Viability Assessment: A fluorescence-based LIVE/DEAD® viability/cytotoxicity assay kit consisting of intracellular green-fluorescent calcein acetoxymethyl and red-fluorescent ethidium homodimer-1 stains was used to determine the viable and non-viable cells. Briefly, a solution containing calcein (2 µmol L^{-1}) and ethidium homodimer-l (8 µmol L^{-1}) were dissolved in PBS, respectively. At each time point, fibers in the strainer and the media were removed from well-plates and cells were washed with DPBS and subsequently 1 mL of the solution was added to each sample. The samples were then incubated for 15 min at 24 °C and imaged using an inverted fluorescence microscope (λ_{ex}=488 nm). NIH-3T3 cells were passaged every three days (confluency of 70%) before performing the assay, and no cell passage was performed during the biocompatibility tests.

Cell Proliferation Assay: Proliferation of cells was measured using a resazurin-based PrestoBlue® assay. Briefly, NIH-3T3 fibroblasts were seeded in 6-well plate substrates and cultured for 1, 3, and 7 days. At each time point, the culture medium and samples in the strainers were removed and cells were washed with DPBS. Subsequently, the cell media containing PrestoBlue® reagent (10 vol%) was added to each well and incubated at 37 °C for

1 h. The fluorescence of the reduced dye was read at 570 (λ_{ex}) and 600 nm (λ_{em}) with a microplate reader, and values were corrected based on a blank controls (the well with reagent and no cells served as the blank control). Six replicates were analyzed continuously for 1, 3 and 7 days and growth were plotted based on the mean ± standard deviation.

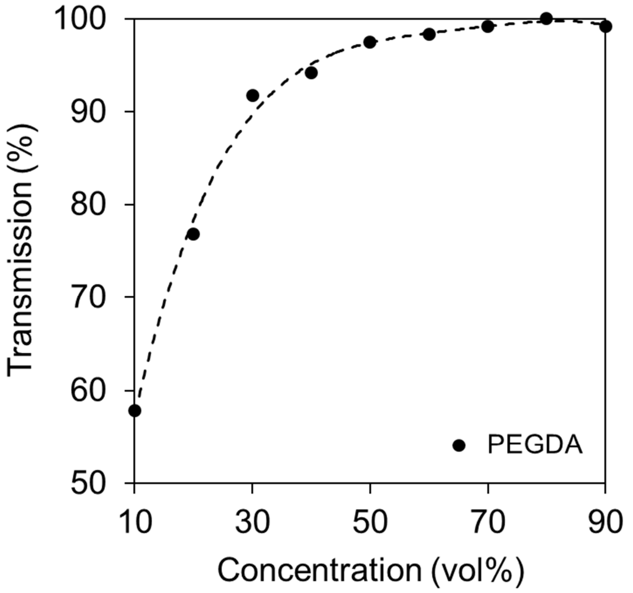

Figure S1. Light transmission through PEGDA (1×1×1 cm^3) at 532 nm

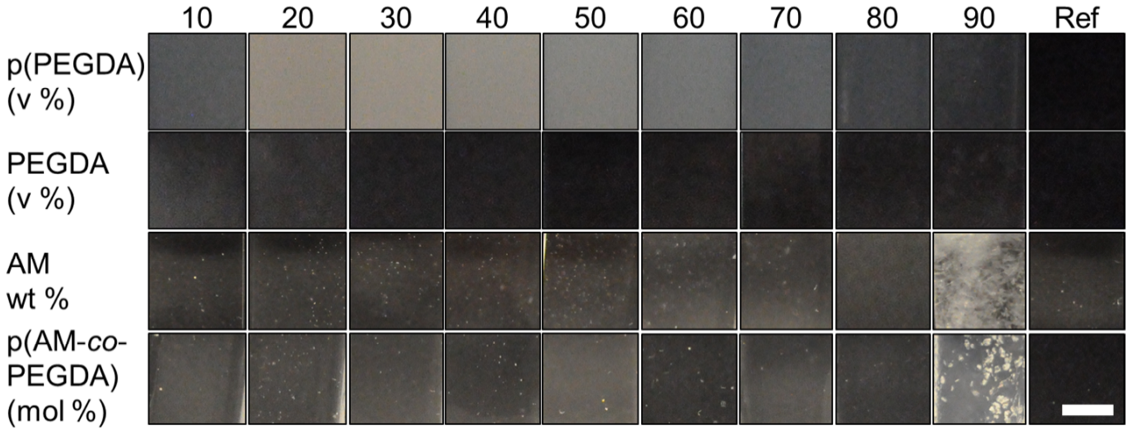

Figure S2. Transparency of hydrogels in cuvettes as a function of precursor concentration. Scale bar = 5 mm.

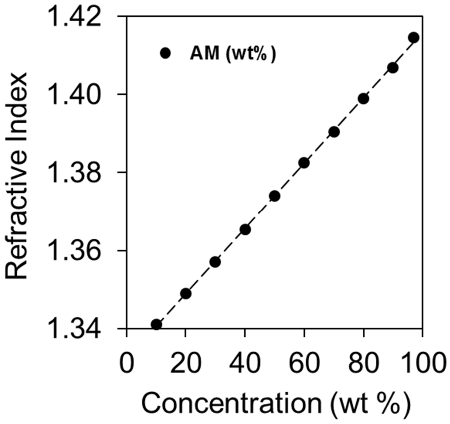

Figure S3. Refractive index of acrylamide

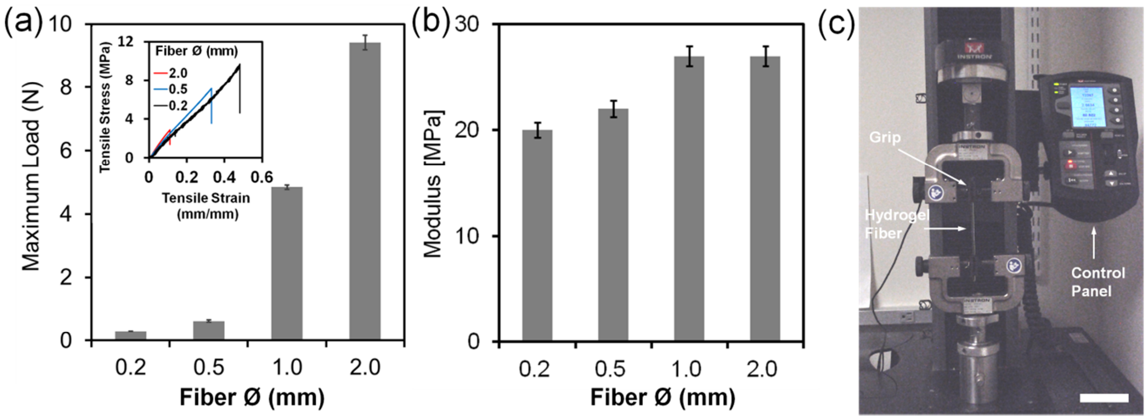

Figure S4. Mechanical measurements of the hydrogel optical fibers. (a) Maximum load of p(PEGDA) fibers (80 vol%). Insets shows tensile stress of p(AM-*co*-PEGDA) hydrogel fibers with different diameters. (b) Modulus of p(AM-*co*-PEGDA) hydrogel fibers with different diameters. Error bars represent three independent samples (n=3). (c) Experiment setup for measuring the tensile stress in Instron analyzer. Scale bar = 5 cm.

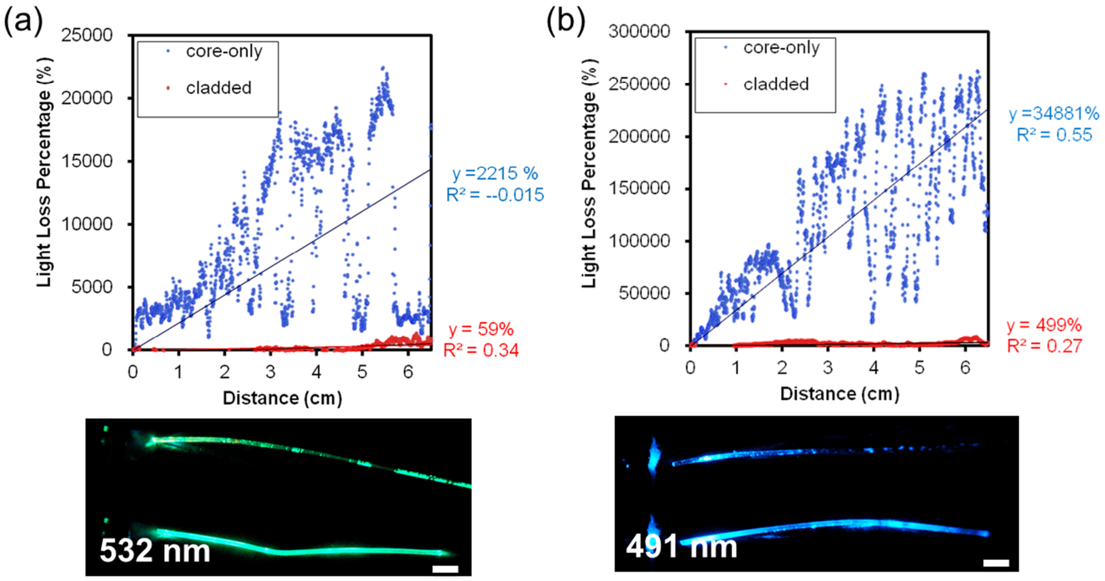

Figure S5. Light loss through hydrogel optical fibers using laser light at (a) 532 nm and (b) 491 nm. Scale bars= 10 mm.

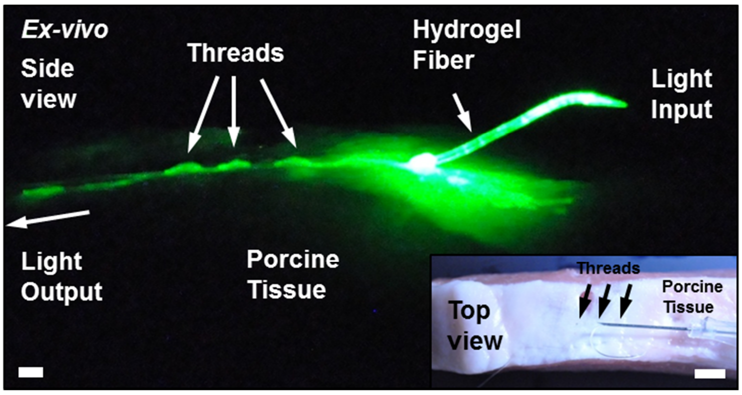

Figure S6. Hydrogel fibers implanted into porcine tissue *ex vivo*. Hydrogel fibers sawn into porcine tissue *ex vivo*. Side view of the hydrogel fiber. Scale bar = 1 mm. Inset shows the top view of the hydrogel fiber sawn into the tissue. Scale bar = 10 mm.

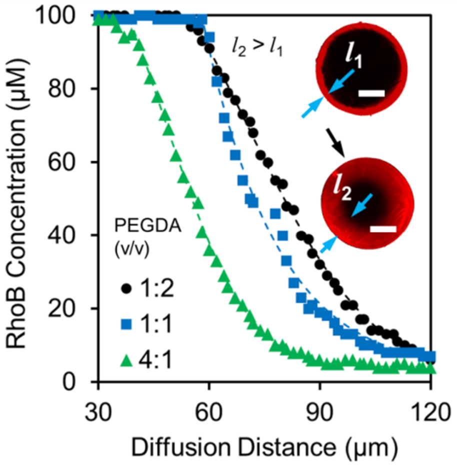

Figure S7. The diffusion of rhodamine B into hydrogel optical fibers. Inset shows the fluorescent images of hydrogel fiber cross-sections. Scale bar = 750 μm.

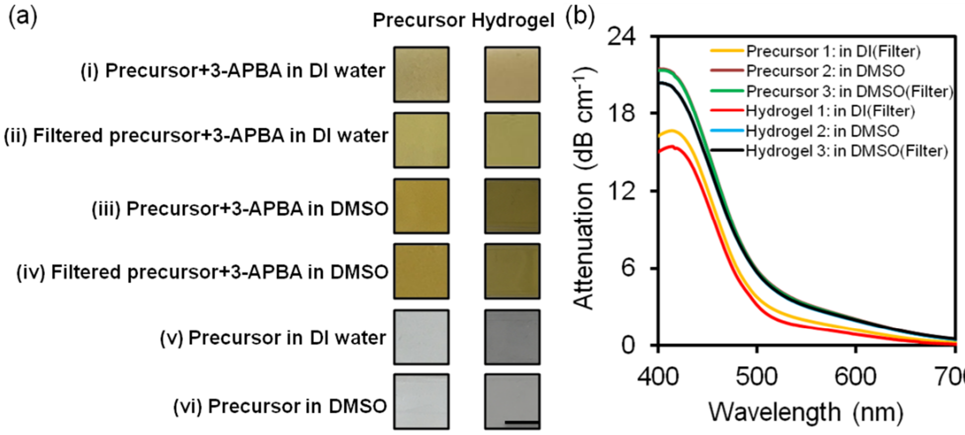

Figure S8. Characterization of precursors and hydrogels in DI and DMSO before and after filtering (0.22 μm) at 24°C. (a) Photographs of the precursors and hydrogels in cuvettes. Precursors(AM:PEGDA:3-APBA, 77/3/20 mol%) and resulting hydrogels (i) in DI water; (ii) filtered in DI water; (iii) in DMSO; (iv) Filtered in DMSO; (v) Precursor and hydrogel (AM:PEGDA, 97/3 mol% in DI water); (vi) Precursor and hydrogel (AM:PEGDA, 97/3 mol% in DMSO). The sample consisting of the precursor (i) was turbid before filtration. Scale bar: 0.5 μm. (b) Spectrophotometry results of the samples.

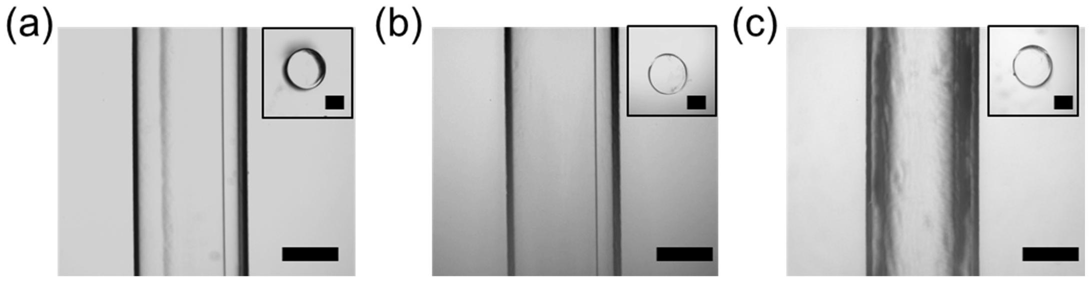

Figure S9. Hydrogel fiber expansions in different concentrations of glucose solutions. (a) 0 mmol L^{-1}, (b) 10 mmol L^{-1}, (c) 20 mmol L^{-1}. The insets are the corresponding cross-section images. Scale bar= 0.5 mm.

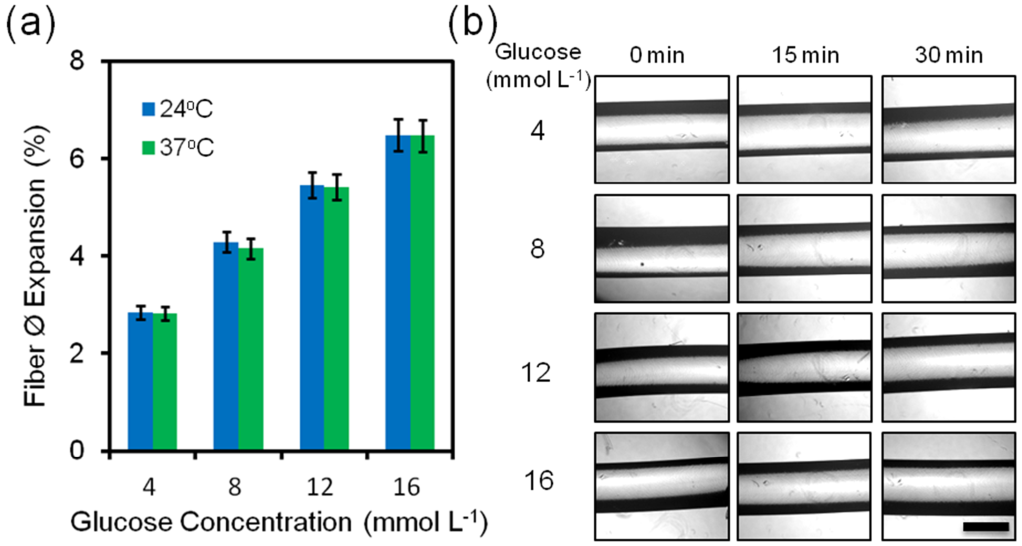

Figure S10. Diameter expansions of p(AM-*co*-PEGDA-*co*-3-APBA) hydrogel fiber (Ø=1.0 mm) in glucose solutions in tris buffers (pH 7.4, 100 mmol L^{-1}) at 24 °C and 37 °C. (a) Quantification of hydrogel fiber diameter expansion within physiological glucose concentrations (4-16 mmol L^{-1}) (n=3); (b) Corresponding optical microscope images of hydrogel fiber expansion. Scale bar= 1.0 mm.

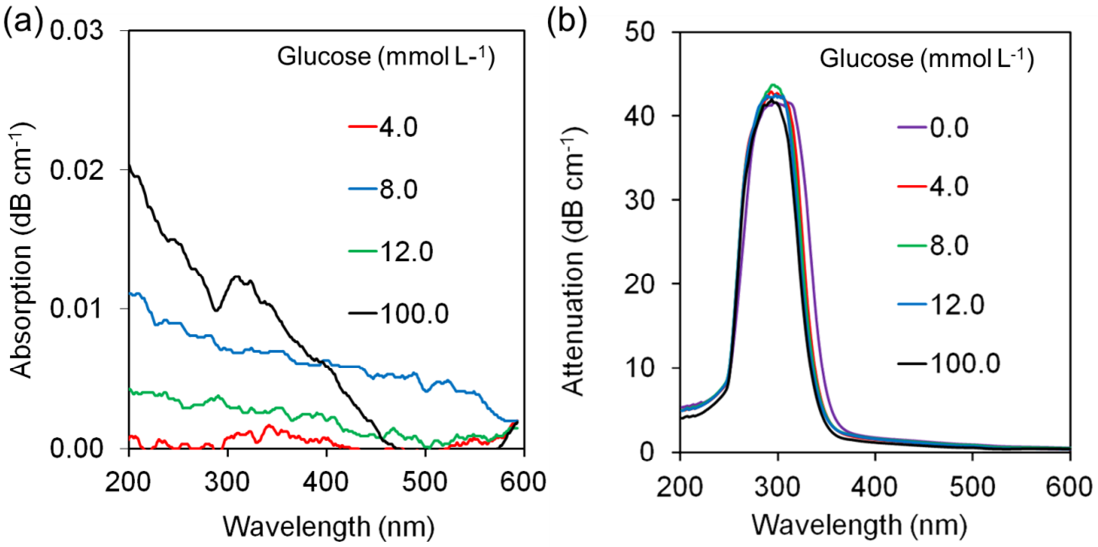

Figure S11. Absorption of light by glucose in the physiological range. (a) Glucose solutions in DI water. (b) Glucose in p(PA-*co*-PEGDA-*co*-3-APBA) (82:3:15 mol%)

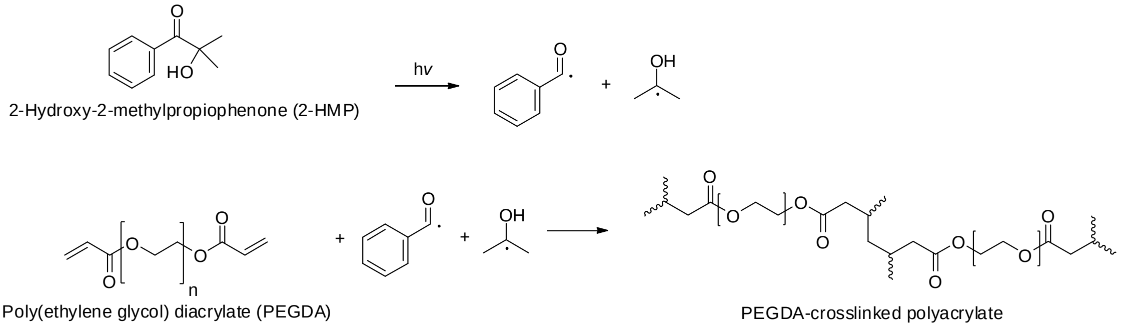

Scheme S1. Synthetic scheme of PEGDA-crosslinked polyacrylate synthesis

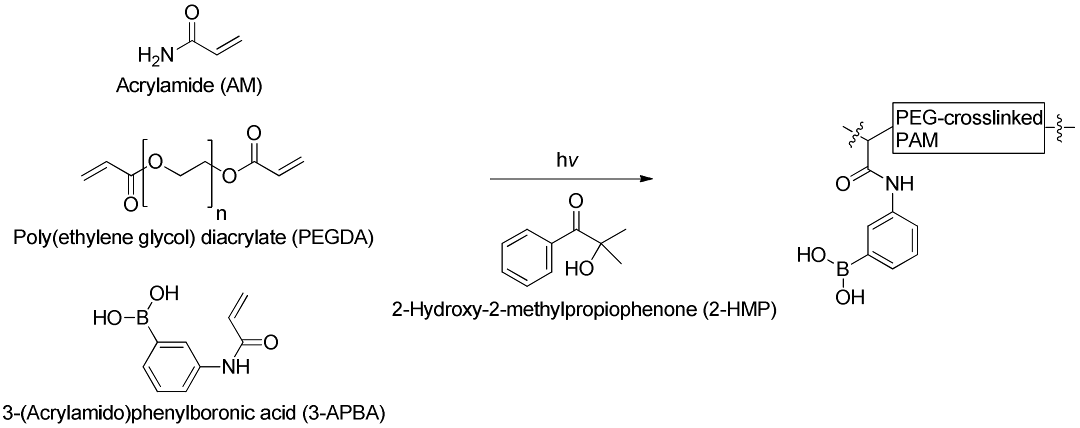

Scheme S2. Synthetic scheme of PAM-*co*-PEG-3-APBA

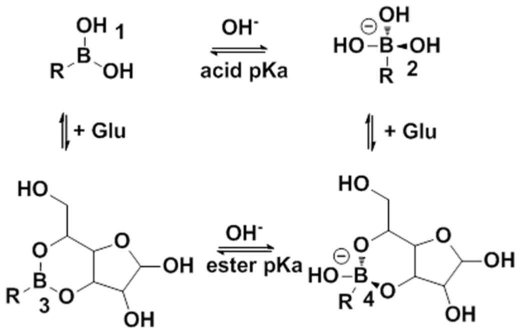

Scheme S3. Complexation of 3-APBA with a cis diol of glucose molecule. (1) uncharged, trigonal planar form, (2) tetrahedral state, (3) strained complex with glucose, and (4) predominant complex with tetrahedral state

Table S1. Commercial sensors such as Dexcom 4G utilize a 26-gauge (OD: 464 µm, ID: 260 µm) for the insertion of glucose sensor. These sensors are inserted with a 45° angle into the adipose tissue. The table below shows the dimensions of the commercial sensors as compared to the present work. As it can be seen clearly, the present hydrogel fiber is thinner as compared to the commercial products in the market.

**Product**	**Sensor Length (mm)**	**Sensor diameter at the base (µm)**	**Sensor diameter at the end (µm)**
Dexcom Seven® Plus	13	400	300
Dexcom G4™	13	250	250
Present work	9-14	200	200
